# Explaining the effects of two different strategies for promoting hand hygiene in hospital nurses: a process evaluation alongside a cluster randomised controlled trial

**DOI:** 10.1186/1748-5908-8-41

**Published:** 2013-04-08

**Authors:** Anita Huis, Gerda Holleman, Theo van Achterberg, Richard Grol, Lisette Schoonhoven, Marlies Hulscher

**Affiliations:** 1 Scientific Institute for Quality of Healthcare, Radboud University Nijmegen Medical Centre Nijmegen, Nijmegen, The Netherlands; 2 Faculty of Health Sciences, University of Southampton, Southampton, UK

**Keywords:** Process evaluation, Quality improvement, Implementation, Handwashing, Infection control, Randomised controlled trial, Leadership

## Abstract

**Background:**

There is only limited understanding of why hand hygiene improvement strategies are successful or fail. It is therefore important to look inside the ‘black box’ of such strategies, to ascertain which components of a strategy work well or less well. This study examined which components of two hand hygiene improvement strategies were associated with increased nurses’ hand hygiene compliance.

**Methods:**

A process evaluation of a cluster randomised controlled trial was conducted in which part of the nursing wards of three hospitals in the Netherlands received a state-of-the-art strategy, including education, reminders, feedback, and optimising materials and facilities; another part received a team and leaders-directed strategy that included all elements of the state-of-the-art strategy, supplemented with activities aimed at the social and enhancing leadership. This process evaluation used four sets of measures: effects on nurses’ hand hygiene compliance, adherence to the improvement strategies, contextual factors, and nurses’ experiences with strategy components. Analyses of variance and multiple regression analyses were used to explore changes in nurses’ hand hygiene compliance and thereby better understand trial effects.

**Results:**

Both strategies were performed with good adherence to protocol. Two contextual factors were associated with changes in hand hygiene compliance: a hospital effect in long term (p < 0.05), and high hand hygiene baseline scores were associated with smaller effects (p < 0.01). In short term, changes in nurses’ hand hygiene compliance were positively correlated with experienced feedback about their hand hygiene performance (p < 0.05). In the long run, several items of the components ‘social influence’ (i.e., addressing each other on undesirable hand hygiene behaviour p < 0.01), and ‘leadership’ (i.e., ward manager holds team members accountable for hand hygiene performance p < 0.01) correlated positively with changes in nurses’ hand hygiene compliance.

**Conclusion:**

This study illustrates the use of a process evaluation to uncover mechanisms underlying change in hand hygiene improvement strategies. Our study results demonstrate the added value of specific aspects of social influence and leadership in hand hygiene improvement strategies, thus offering an interpretation of the trial effects.

**Trial registration:**

The study is registered in ClinicalTrials.gov, dossier number: NCT00548015.

## Background

Strategies to improve adherence to practice guidelines are often multimodal and consist of a number of potentially effective components and related improvement activities [1-3] (See Table 1). All of these components might influence effectiveness both independently and inter-dependently. This poses challenges for strategy evaluation. A randomised controlled trial (RCT) is the most rigorous way to evaluate the effectiveness of improvement strategies, regardless of their complexity. However, published reports of RCTs mainly focus on the outcomes, answering the question ‘Does it work?’ [4,5]. RCTs rarely answer the question of why an improvement strategy has been successful or has failed [6]. Despite the CONSORT guidelines [7], a detailed description of an improvement strategy — reporting on all components and corresponding activities — and how well the strategy was performed is often lacking. This equally applies to information on contextual aspects such as the environment or setting, as well as factors that inhibited or promoted effectiveness [4,8]. Understanding RCT results is also complicated by the use of intention-to-treat analyses. To provide unbiased comparisons among the treatment groups, individuals or clusters are analysed according to the group (experimental or control) to which they were originally allocated, regardless of whether they actually received the improvement strategy. Therefore, it is necessary to combine the strength of an RCT with a well-designed process evaluation [9].

**Table 1:** Explanation of terms used in this article

**Term**	**Explanation**
**Hand hygiene improvement strategy**	An HH improvement strategy is composed of a number of components intended to change HH behaviour. These various components work best together and support each other in targeting potential barriers to appropriate HH.
**Strategy component**	A strategy component refers to the specific method used to address a potential barrier to appropriate HH.
	Examples: education, reminders, performance feedback, social influence, leadership, setting norms and targets.
**Improvement activities**	Improvement activities refer to the operationalization of strategy components.
	Examples: educational website, bar charts of HH rates, posters, ward manager addresses barriers to enable HH as recommended, provision of alcohol-based hand rub.
**Intention**-**to**-**treat analysis**	The intention-to-treat analysis in our study was an analysis based on the initial treatment intent. In this, wards were analysed according to the group (experimental or control) to which they were originally allocated, regardless of whether they actually received the improvement strategy and despite the fact that there may be less impact on those who did not receive the intervention
**As**-**received analysis**	The as-received analysis in our study is based on the treatment actually received. In this, wards were analysed according to improvement strategy actually received, regardless of their allocation.

Process evaluations are important because they can clarify to what extent the improvement strategy was performed in a uniform way, whether the target population actually received the planned activities, what factors inhibited or promoted effectiveness, and what the participants’ actual experiences with the executed strategy were [5,10-12]. Process evaluations also provide information important to understanding and validating theory-informed strategies. Identifying the mechanisms for how and why these strategies produce successful change (or fail to produce change) is crucial to refining theory and improving strategy effectiveness [13].

Combined analysis of process and outcome data allows evaluations to explore associations between strategy delivery and receipt, and outcomes on effectiveness [14]. In this way, insight is gained into the mechanisms responsible for the results, which could improve the validity of the findings and help researchers understand the potential generalizability of the improvement strategy [10,11,15].

### The case of hand hygiene: the HELPING HANDS study

Hospital acquired infections are the most common complications in hospital care, and a major threat to patient safety [3,16]. Hand hygiene (HH) is considered the most important measure in the prevention of hospital acquired infections [3,17,18]. Unfortunately, compliance with HH recommendations is repeatedly found to be insufficient [3,17,18]. Many potentially effective strategies for improving HH compliance are described, but most of the effects are small to moderate [2,19,20]. Traditionally, strategies have concentrated on the healthcare professional or focused on the introduction of new products and facilities [2,20]. However, often experienced barriers like negative role models, lack of management involvement, and poor social culture are rarely addressed [21]. Using insights from the behavioural sciences and performing a strategy that also targets social influence within teams and leadership could be a valuable addition to HH implementation strategies [21-23].

In a recent study, we undertook a cluster randomised trial (the HELPING HANDS study) at 67 nursing wards in three Dutch hospitals to compare the effectiveness of a state-of-the-art strategy with a team and leaders-directed strategy for improving nurses’ compliance with HH guidelines [24,25]. The state-of-the-art strategy was based on current evidence from literature on HH compliance [3,16,20]. This strategy targeted the individual and organizational level and included the following components: education for improving relevant knowledge and skills; reminders for supporting the actual performance of HH; feedback as a means to provide insight into current HH behaviour and to reinforce improved behaviour; and screening for adequate HH products and adequate facilities. The team and leaders-directed strategy was also aimed at addressing barriers at team-level by focussing on social influence within teams and strengthening leadership of the ward manager. The unique contribution of this strategy was built upon the social learning theory [26], social influence theory [27], theory on team effectiveness [28,29], and leadership theory [30]. Together, these theories provide a coherent set of methods to target the social context in which HH behaviour takes place. Table 2 provides an overview of our theory selecting process, including the characteristics and key elements of the behaviour change theories. The identified key elements were used to build our team and leaders-directed strategy that included all components of the state-of-the-art strategy, supplemented with: gaining active commitment and initiative of ward management; modelling by informal leaders at the ward; and setting norms and targets within the team. Before the start of the intervention, all managers participating in the team and leaders-directed group received a four-hour training in coaching and motivating the nurses. During the intervention period, the ward manager was assisted by an experienced coach in three team meetings. Also, two group sessions were organised to support the ward managers and to discuss progress and difficulties. Table 3 presents the content and related activities of both strategies.

**Table 2:** Selected behaviour change theories matching barriers in performing HH

**Theory**	**Focus**	**Key elements**
Social learning theory [26]	Behaviour is learned from the environment through the process of observational learning.	– Demonstration, role modelling.
		– Encompasses attention, memory, and motivation.
Social influence theory [27]	Social norm in a network determines what correct behaviour is.	– Norm and target setting.
		– Commitment team members.
		– Use of opinion leaders.
		– Performance feedback.
		– Team members address each other in case of undesirable behaviour.
Theory on team effectiveness [28,29]	Orientation on team climate and willingness to change	– Team Vision: clarity, perceived value, and attainability.
		– Participation Safety: decision-making, information sharing, interaction and safety.
		– Support for Innovation: articulated and enhanced support.
		– Task Orientation: commitment to excellence, appraisal and task orientation.
Theories of leadership [30]	Leading, coaching and managing a team	– Active commitment/participation in performance improvement initiatives.
		– Setting norms and targets/direction/expectations.
		– Encouragement and support/motivate staff.
		– Monitoring performance and feedback.

**Table 3:** Description of the implementation strategies with the planned activities

**State-of-the-art strategy**	**Team and leaders-directed strategy**
**Education**	**All elements of the state-of-the-art strategy**
Distribution of educational material/ written information (leaflet) about HH that contained:	• Education, reminders, feedback, facilities and products, see above
• The importance of HH	**Setting norms and targets within the team**
• Misconceptions about alcohol-based HH disinfection	• Three interactive team sessions (1 h-1.5 h each) that included goal setting in HH performance at group level. Team sessions were guided by the team manager and a external coach.
• Theory and practical indications for the use of HH	**◦** Exploring nurses’ knowledge and perception of current HH behaviour (individual- and team level) and discussing actual HH compliance rates
Website http://www.gewoonhandenschoon.nl	**◦** Transition from individual responsibility to a shared team responsibility
• Educational material/ written information about HH	**◦** Creating a participatory and non-threatening climate for team interaction
• Knowledge quiz with feedback. Visitors could test their knowledge about HH	**◦** Commitment to high standards of HH performance
• The nursing ward with the highest number of visitors to the website was rewarded	**◦** Defining and documenting improvement activities
Educational sessions on prevention of hospital acquired infections	• Analysis of barriers and facilitators to determine how nurses could best adapt their behaviour in order to reach their goal.
• Launching hospital-wide campaign with practical demonstrations of HH	• Nurses address each other in case of undesirable HH behaviour
**Reminders**	**Gaining active commitment and initiative of ward management**
• Distribution of posters that emphasised the importance of HH, particularly alcohol-based hand disinfection. Posters were displayed in several strategic areas within the units and replaced by another poster after 12 weeks.	• Ward manager designated HH as a priority
• Interviews and messages in newsletters or hospital magazines	• Ward manager actively supported team members and informal leaders
• General reminders by opinion leaders/ ward management	• Ward manager discussed HH compliance rates with team members
**Feedback**	**Modeling by informal leaders at the ward**
• Bar charts of HH rates of every nursing ward were sent to the ward manager twice. This also included a comparison of ward performance and hospital performance	• Informal leaders demonstrated good HH behaviour
**Facilities and products**	• Informal leaders modeled social skills of team members in addressing HH behaviour of colleagues
• Screening and if necessary adapt products and appropriate facilities	• Informal leaders instructed and stimulated their colleagues in providing good HH behaviour

Both strategies successfully improved hand hygiene compliance, but the team and leaders-directed strategy showed better results [24]. The findings of this study indicated the added value of strategy components aimed at social influence within teams and enhanced leadership of ward managers on nurses’ HH behaviour. However, these results provide no insight into the mechanisms of impact. For instance, the extent to which nursing wards improved their HH compliance varied considerably for both strategies, ranging from −2% to 70% improvement in the long run. In addition, the effect size of the team and leaders-directed group was limited by the intention-to-treat analysis, which is the main statistical approach for RCT analyses. Wards were analysed according to the group state-of-the-art strategy or team and leaders-directed strategy to which they were originally allocated. In the HELPING HANDS study, thirty nursing wards and their managers were randomly assigned to the team and leaders-directed group but ten wards declined to participate in the team and leaders-directed strategy. Therefore, only twenty wards fully participated in the team and leaders-directed group.

The current article expands on the findings of the HELPING HANDS study by linking process and effectiveness evaluations. The aim of this paper is to ascertain which components of the two HH improvement strategies can be particularly associated with increased nurses’ HH compliance, as well as to explore other possible factors that may be associated with changes in nurses’ HH compliance. We focused on three specific questions:

1. What impact might variation in adherence to the improvement strategies as planned have on changes in nurses’ HH compliance?

2. What impact might specific contextual factors as hospital and ward characteristics have on changes in nurses’ HH compliance?

3. What impact might differences in nurses’ actual experiences with strategy components have on changes in nurses’ HH compliance?

## Methods

### Setting and participants

The HELPING HANDS study was performed in three hospitals in the Netherlands: two general hospitals and one university medical centre. Within the hospitals, all in-patient nursing wards (n = 67) and all affiliated nurses participated in the study. We included surgical wards (n = 21), internal medicine wards (n = 24), intensive care units (n = 13), and paediatric wards (n = 9). Twenty wards received the team and leaders-directed group, and 47 wards received the state-of-the-art group. Strategies were delivered during a period of six months. Follow-up measurements took place directly after strategy delivery (T2) and at six months after the end of strategy delivery (T3).

### Measurements and data collection

Data were collected using a wide range of methods, including: student observations, questionnaires to nurses, a ward structure survey, registration of website visitors, structured logbooks of ward managers and coaches and researchers’ field notes of group meetings. Using these data sources, we constructed four sets of measures.

### Effect evaluation

#### Effects on nurses’ HH compliance

The primary outcome was the percentage of nurses’ actions in line with HH guidelines in case of an opportunity to perform this action, according to the HH guidelines of the World Health Organization [3,31]. We monitored nurses’ HH compliance unobtrusively during routine patient care before and directly after strategy delivery, as well as six months later [24].

### Process evaluation

#### Adherence to the improvement strategies as planned

The measurement of adherence captures the following subcategories: content – whether improvement activities were delivered as planned (yes/no); dosage – whether improvement activities were delivered as often and long as planned (yes/no); coverage – the extent to which the intended target group received the improvement activities [32].

Education was assessed by monitoring the presence of instruction leaflets on the ward and by measuring the number of nurses who completed the knowledge quiz. The use of reminders was checked by measuring the presence of reminders (posters) at random moments during the strategy delivery period. Feedback was assessed by checking the distribution of performance feedback reports to ward managers and by a question from the study’s survey asking if nurses had received performance feedback from the ward manager. In addition, the extent to which products and facilities were available in each ward was also explored by survey questions to ward managers and nurses. The attendance of ward management and informal leaders to the training sessions and the support sessions was derived from an attendance checklist. The use of coaching of ward management and informal leaders was assessed by measuring the number of coaching sessions and the total time spent on coaching. Additional details on coaching activities are available from the authors on request. The use of organised team discussions for norm- and target-setting was checked by measuring the number of team discussions performed, the number of nurses attending per ward, the time investment per ward, and whether norms and targets were established. Leadership was assessed by checking documented agreements on the following points: whether the ward manager had discussed HH compliance rates during the team sessions; whether the ward manager had prioritized good HH as a ward target; and whether the ward manager had formulated specific activities to support the team members and informal leaders. Finally, information on whether informal leaders served as role models was derived from group discussion during the support sessions for ward managers and informal leaders.

### Contextual factors

We explored the influence of three contextual variables, namely: hospital, ward specialism (e.g., general ward, surgical ward, paediatric ward or critical care ward) and the HH compliance rate at baseline.

### Nurses’ experiences with specific components of the improvement strategies

In order to explore the relationship between HH outcomes and nurses’ actual experiences with different strategy components, we drew on the findings of a 7-subscale questionnaire consisting of 24 items. Each item was a proposition on a specific component of the improvement strategies. These components were education, reminders, feedback, facilities and products, setting norms and targets, social influence and leadership. An example of a proposition that explores nurses’ actual experiences with leadership is ‘my ward manager holds team members accountable for HH performance.’ Nurses scored this proposition on a 4-point Likert scale, ranging from strongly agree (4) to strongly disagree (1). Negatively formulated propositions were recoded. Higher scores indicated more positive experiences with respective components (Additional file 1).

### Statistical analyses

In this study, our primary research goal was to understand the working mechanisms of HH improvement strategies embedded in the relationship between strategy performance and nurses’ HH compliance. Therefore, we combined data from the process evaluations with data from the effect evaluation. To serve our research goal, we moved from the original intention-to-treat analysis to an as-received basis, with 47 wards in the state-of-the-art group and 20 wards in the team and leaders-directed group. Inputs for the effect analysis, used in this paper, were based on the HH compliance findings of the previously mentioned HELPING HANDS study. The effectiveness of the HELPING HANDS study was examined using an ‘intention-to-treat’ analysis. However, 10 wards declined to participate in the team and leaders-directed group and did not receive any component of this strategy. We therefore explored whether the inclusion, in our intention-to-treat analysis, of wards who did not receive the team and leaders-directed strategy, might have resulted in different effects in changes in nurses’ HH compliance. All data were analysed using SPSS version 19.0 (SPSS, Inc., Chicago, IL) and analyses were performed at ward level.

### Effect evaluation

#### Effects on nurses’ HH compliance: intention-to-treat versus as-received analysis

We compared the outcome data on changes in HH compliance of the intention-to-treat analysis (37 wards in the state-of-the-art group and 30 wards in the team and leaders-directed group) with the results of the as-received analysis (47 wards in the state-of-the-art group and 20 wards in the team and leaders-directed group). We used descriptive statistics, including mean and standard deviation, for the change in HH compliance between the measurement points for each of the two strategies. One-way ANOVAs were used to test whether there was a statistically significant difference between the group means for both strategies. A p value of 0.05 or less was considered to indicate the statistical significance of the difference between measurements at baseline (T1), directly after strategy delivery (T2), and at six months after the end of strategy delivery (T3).

### Process evaluations linked to effectiveness evaluations

#### Analysis of adherence to the improvement strategies and related changes in HH compliance

Frequencies and proportions were used to assess the adherence to the several components of the improvement strategies. One-way ANOVAs were used to test the influence from varying strategy components on HH compliance. If a strategy component was significant, correlations between changes in nurses’ HH compliance and the significant term were also examined within each strategy group using the Spearman correlation analysis.

### Analysis of contextual factors and related changes in HH compliance

One-way ANOVAs were used to test the influence from the contextual factors hospital, ward specialism, and the HH compliance rate at baseline. The correlation between nurses’ HH baseline scores and changes in nurses’ HH compliance was tested with the Pearson correlation analysis. Next, we applied forced entry multiple regression analyses to assess the impact of several potential explanatory variables on changes in HH compliance. As an estimation for the explained variance of the model, an adjusted R-Squired was determined.

### Analysis of nurses’ actual experiences with specific components of the improvement strategies and related changes in HH compliance

Descriptive statistics, including mean and standard deviation, were used to explore differences in nurses’ actual experiences with specific strategy components between nurses in the team and leaders-directed group, and in the state-of-the-art group. Inclusion criteria for analysis were wards whose respondents returned ≥ 3 questionnaires. One-way ANOVAs were used to test whether there was a statistically significant difference between group means for both strategies. To determine whether differences in nurses’ actual experiences with strategy components predicted variation in HH compliance effects, we tested non-parametric correlations with Spearman analyses between groups and within groups.

## Results

### General

Initially, 67 wards were included, 30 to the team and leaders-directed group, and 37 to the state-of-the-art group. Ten wards declined to participate in the team and leaders-directed group because of a vacancy for the position of ward manager (2×), reorganization of the ward (2×), workload of the ward manager ruled out other activities (1×), inconvenient timing relating to the execution of the strategy (2×), or other projects were given a higher priority (3×). Finally, 47 wards received only the state-of-the-art strategy, and 20 wards received the team and leaders-directed strategy (Table 4). At each point in time, 3,523 to 3,722 opportunities for HH were observed in 886 to 933 nurses. During the entire study, we obtained data on 10,785 opportunities for HH in 2733 nurses.

**Table 4:** Characteristics of the wards

**Ward characteristics**	**SAS**^**†**^	***n=47***	**TDS**^**‡**^	***n=20***
*Hospital*	University based hospital	*n=16*	University based hospital	*n=9*
	General hospital A	*n=15*	General hospital A	*n=5*
	General hospital B	*n=16*	General hospital B	*n=6*
*Specialism*	Surgical ward	*n=14*	Surgical ward	*n=7*
	Medical ward	*n=16*	Medical ward	*n=8*
	Intensive care unit	*n=12*	Intensive care unit	*n=1*
	Paediatric ward	*n=5*	Paediatric ward	*n=4*

^†^State-of-the-art strategy.

^‡^Team and leaders-directed strategy.

### Effect evaluation

#### Effects on nurses’ HH compliance: intention-to-treat versus as-received analysis

Table 5 displays the results of changes in nurses’ HH compliance derived from the intention-to-treat analysis and the as-received analysis. In both analyses, the team and leaders-directed group demonstrated better results on HH compliance than the state-of-the-art group. The as-received analysis showed higher effect sizes for the team and leaders-directed group than the intention-to-treat analysis. A statistically significant (p = 0.002) increase in nurses’ HH compliance was observed in the long run (T3) in favour of the team and leaders-directed strategy. The intention-to-treat analysis showed no significant difference in nurses’ HH compliance between both strategies at T3.

**Table 5:** Changes in HH compliance in participating hospitals during study period

**Intention-to-treat analysis**	**T1**	**T2**	**T3**
	**baseline**	**post intervention**	**follow-up**
Strategy SAS^†^	21.8% (37 wards)	40.4% (37 wards)	45.9% (37 wards)
		Δ T1-T2 18.6%	Δ T1-T3 24.1%
Strategy TDS^‡^	19.1% (30 wards)	53.1% (30 wards)	52.1% (30 wards)
		Δ T1-T2 34.0%	Δ T1-T3 33.0%
Groups compared			
TDS vs. SAS	*f*=0.465	*f*=19.409	*f*=1.781
ANOVA	*p*=0.498	*p*=0.000**	*p*=0.187*
**As-received analysis**	**T1**	**T2**	**T3**
	baseline	post intervention	follow-up
Strategy SAS^†^	21.5% (47 wards)	40.7% (47 wards)	44.1% (47 wards)
		Δ T1-T2 19.2%	Δ T1-T3 22.6%
Strategy TDS^‡^	20.7% (20 wards)	58.6% (20 wards)	59.5% (20 wards)
		Δ T1-T2 37.9%	Δ T1-T3 38.8%
Groups compared			
TDS vs. SAS	*f*=0.001	*f*=40.304	*f*=10.187
ANOVA	*p*=0.978	*p*=0.000**	*p*=0.002**
Groups compared			
SAS groups randomised to TDS (*n*=10) vs SAS groups randomised to SAS (*n=*37)	*p*=0.322	*p*=0.650	*p*=0.224
T-test			

Compliance with HH prescriptions expressed as a percentage of all relevant opportunities based on the average compliance per ward.

^†^State-of-the-art strategy.

^‡^Team and leaders-directed strategy.

*p < .05; **p < .01.

### Process evaluations linked to effectiveness evaluations

#### Adherence to the improvement strategies and related changes in HH compliance

Both improvement strategies were carried out with good adherence to protocol. Detailed results on strategy adherence are described in Additional file 2.

##### Impact of variation in adherence to the components of the state-of-the-art strategy (n = 67)

In the adherence subcategory ‘content,’ we found that the main components of the state-of-the-art strategy were generally delivered as planned. The ‘HH promotion event’ was not delivered in one hospital. The infection control department of this particular hospital had already organised an HH promotion event one year before the start of our study. Despite the variation in delivering the ‘HH promotion event,’ no effect on changes in HH compliance could be demonstrated (p = 0.384). The subcategory ‘coverage’ showed some variation in the extent to which washstands were accessible. The analysis showed that variation within these components had no effect on changes in HH compliance (p = 0.348).

The subcategory ‘coverage’ also demonstrated a significant difference between the number of nurses from wards receiving the state-of-the-art strategy and the number of nurses from wards receiving the team and leaders-directed strategy in completing the knowledge quiz (13% and 37%; p = 0.001). This was positively correlated with changes in HH compliance at both follow-up measurements (T1 to T2: p = 0.019; T1 to T3: p = 0.016) However, completing the knowledge quiz did not predict variation in HH compliance within groups of the state-of-the-art strategy (T1 to T2: p = 0.779; T1 to T3: p = 0.426) or within groups of the team and leaders-directed strategy (T1 to T2: p = 0.354; T1 to T3: p = 0.452).

##### Impact of variation in adherence to the additional components of the team and leaders-directed strategy (n = 20)

In the adherence subcategory ‘content,’ we found that all components of the team and leaders-directed strategy were delivered as planned. Components that differed in adherence across the wards concerned the subcategories ‘dose’ and ‘coverage.’ Five wards organised only two team sessions instead of three team sessions. Thus, these wards did not receive a full dose. However, this did not affect the course of nurses’ HH compliance (T1 to T2: p = 0.240; T1 to T3: p = 0.254). Full coverage was also not achieved for attending two sessions in support of the role models and ward managers, but everyone took part in at least one session. Variation in adherence within the component ‘support sessions’ had no effect on changes in HH compliance (ward managers T1 to T2: p = 0.262; T1 to T3: p = 0.994; role models T1 to T2: p = 0.184; T1 to T3: p = 0.688). There was also some variation in the average number of nurses that attended the team sessions, related to total number of nurses employed. However, variation within this component had no effect on changes in HH compliance (T1 to T2: p = 0.445; T1 to T3: p = 0.823). In conclusion, the evaluation of strategy adherence did not provide any explanatory variables associated with changes in nurses’ HH compliance.

### Contextual factors and related changes in HH compliance

Our next step was to determine the impact of contextual factors on changes in nurses’ HH compliance. Two contextual factors were associated with changes in HH compliance: type of hospital and HH performance at baseline. The ANOVA showed a hospital effect on changes in HH compliance in long term (p = 0.036). HH compliance decreased in one hospital in long term, while the HH compliance in the other two hospitals remained stable or increased further. At baseline, the HH scores of all wards from the state-of-the-art strategy and the wards that participated in the team and leaders-directed group were comparable (p = 0.978). For both study groups, baseline HH scores were negatively correlated with follow-up scores (r = −0.693; p = 0.000). Initially, short-term changes in HH compliance (T1 to T2) revealed a specialism effect (p = 0.002). In particular, the paediatric wards showed a smaller increase in HH compliance than the wards from other specialisms. However, the baseline HH scores of the paediatric wards were significantly higher than the baseline HH scores of other wards (p = 0.000). This alleged specialism effect was, in reality, a baseline effect.

We then tested all significant variables in forced entry multiple regression analyses. Table 6 presents the results from two multiple regression analyses. The basic model included baseline HH compliance (covariate), hospital, specialism and strategy. The first model analysed changes in HH scores from baseline (T1) to the first follow-up measurement, directly after strategy delivery (T2). Baseline HH scores (p < 0.01) and hospital (p < 0.05) contributed negatively to short-term changes in HH compliance. The team and leaders-directed strategy contributed positively to short-term changes in HH compliance (p < 0.01). The second model analysed changes in HH compliance from baseline (T1) to the second follow-up measurement, six months after the end of strategy delivery (T3). Baseline HH scores (p < 0.01) and hospital (p < 0.01) contributed negatively to long-term changes in HH compliance. The team and leaders-directed strategy contributed positively to long-term changes in HH compliance (p < 0.01). The adjusted R^2^ was 0.702 for the first model and 0.510 for the second model. This suggests that 70% and 51% of the variation in HH change scores could be explained by the regression model.

**Table 6:** Summary of forced entry multiple regression analysis for variables predicting changes in HH compliance in participating hospitals during study period

	**Δ HH compliance T1 to T2**			**Δ HH compliance T1 to T3**		
**Variable**	**B**	***SE *B**	**β**	**B**	***SE *B**	**β**
Constant	27.78	6.32		47.74	7.78	
Baseline T1	-.91	.94	-.80^**^	-.69	.12	-.64^**^
Strategy	17.29	2.61	.45**	13.47	3.21	.36**
Hospital	-.3.92	1.66	-.19*	-.12.17	2.03	-.60**
Specialism	.72	1.28	.04	.41	1.60	.03
*R*^*2*^		.70			.51	
*F* for change in *R*^*2*^		39.83**			18.18**	

*p < .05; **p < .01.

### Nurses’ experiences with the improvement strategies and related changes in HH compliance

In this section, we explored differences in nurses’ actual experiences with strategy components and how these differences affected changes in nurses’ HH compliance. A total of 528 questionnaires out of 1,100 (369 questionnaires from the state-of-the-art group and 159 from the team and leaders-directed group) were returned, giving a response rate of 48%. Questionnaires of 515 nurses from 59 wards met the inclusion criteria for analysis. Of these, 42 wards belonged to the state-of-the-art group (360 questionnaires), and 17 wards to the team and leaders-directed group (155 questionnaires).

The ANOVA showed significant differences in actual experiences with several items of the questionnaire between nurses from the state-of-the-art group and nurses from the team and leaders-directed group. Nurses from the team and leaders-directed group, who unlike the nurses from the state-of-the-art group were exposed to the strategy components ‘setting norms and targets,’ ‘social influence’ and ‘leadership,’ experienced more social support (p = 0.005), social influence (p = 0.046) and leadership (p = 0.011) with respect to HH performance. In addition, these nurses experienced more priority for HH on their ward (p = 0.009) and experienced more feedback about their HH performance (p = 0.000) than did nurses from the state-of-the-art group.

Table 7 displays nurses’ experiences with components of both improvement strategies and their impact on changes in HH compliance. First, we examined the impact of strategy components in both study groups (n = 67). In short term (T1 to T2) and in the long run (T1 to T3), changes in nurses’ HH compliance were positively correlated with experienced feedback about their HH performance (p < 0.05 and p < 0.01, respectively). In the long run (T1 to T3), two items of the component ‘social influence’ correlated positively with changes in nurses’ HH compliance: addressing each other on undesirable HH behaviour (p < 0.01) and support from colleagues in performing HH (p < 0.01). Also in the long run, five items of the component ‘leadership’ correlated positively with changes in nurses’ HH compliance: regular attention to the adherence of HH guidelines (p < 0.05); designation of HH as ward priority (p < 0.05); addressing barriers to enable HH as recommended (p < 0.05); accountability for HH performance (p < 0.01); and encouraging and motivating team members to perform HH (p < 0.01).

**Table 7:** **Nurses**’ **experiences with strategy components and correlations with changes in HH compliance**

**Correlation with changes in HH compliance in all study groups**		
***Component***	Δ T1 to T2	Δ T1 to T3
**Proposition**	*S* rho (p value)	*S* rho (p value)
*Performance feedback*	.315 (.015*)	.347 (.007**)
I do know my ward’s HH performance.		
*Social influence*		.381 (.003**)
My colleagues support each other in performing HH.		
Our team members address each other in case of undesirable HH behaviour.		.414 (.001**)
*Leadership*		.293 (.025*)
My manager pays regular attention to the adherence of HH guidelines.		
HH is not a priority at our ward.		.261 (.046*)
My ward manager addresses barriers to enable HH as recommended.		.319 (.014*)
My ward manager holds team members accountable for HH performance.		.382 (.003**)
My ward manager encourages and motivates our team members to perform HH.		.352 (.006**)
**Correlation with changes in HH compliance within SAS**^†^		
*Education*	-.315 (.042*)	
I know exactly when to perform HH.		
*Leadership*		.387 (.011*)
My ward manager encourages and motivates our team members to perform HH.		
My ward manager holds team members accountable for HH performance.		.398 (.009**)
*Social influence*		.
Our team members address each other in case of undesirable HH behaviour.		.347 (.025*)

Correlation with changes in HH compliance within TDS^‡^.

No significant correlations between scores on specific items and HH change scores.

^†^State-of-the-art strategy^‡^Team and leaders-directed strategy*p < .05; **p < .01.

Within the state-of-the-art group (n = 47), we found a few correlations between nurses’ experiences with strategy components and changes in HH compliance. In short-term, experienced knowledge of HH indications showed a negative correlation with HH change scores (p < 0.05). In the long term, positive correlations with changes in HH compliance could be demonstrated for one item of social influence, namely: addressing each other on undesirable HH behaviour (p < 0.05). We also found positive correlations with changes in HH compliance for two leadership items: accountability for HH performance (p < 0.01) and encouraging and motivating team members to perform HH (p < 0.05). We found no significant correlations between scores on specific items and HH change scores within the group of the team and leaders-directed strategy (n = 20).

## Discussion

In this article, we examined which components of the HH improvement strategies were particularly associated with increased nurses’ HH compliance, as well as other possible factors that may have influenced nurses’ HH compliance. We therefore linked process and effectiveness evaluations in the analysis of findings from the HELPING HANDS study [24].

### Effect evaluation: intention-to-treat versus as-received analysis

In this article, we have tried to explain the effects of two different HH improvement strategies on changes in nurses’ HH. It is important to recognize that this research goal requires a different view on the treatment effects compared to an evaluation of effectiveness. The outcome suggests that the overall conclusions about the effectiveness of the team and leaders-directed strategy arising from the original intention-to-treat analysis may have underestimated the impact and strength of this strategy. The as-received analysis showed higher effect sizes for the team and leaders-directed group than the intention-to-treat analysis on both measurements points. In the long run, we now observed a statistically significant (p = 0.002) increase in nurses’ HH compliance due to the team and leaders-based strategy. This suggests that the team and leaders-directed strategy might have had a more permanent impact on HH outcomes than shown by the intention-to-treat analysis. This corresponds with the findings of Strange, et al. [36]. Their as-received analysis showed higher odds ratios in decreasing risky sexual behaviour than the original intention-to-treat analysis, thereby suggesting that their peer-led sex education program, if consistently implemented, probably had a greater impact on study outcomes.

### Effects of strategy adherence on nurses’ HH compliance

The evaluation of strategy adherence did not provide any explanatory variables associated with changes in nurses’ HH compliance. Thus, variation in the HH outcomes across the wards could not be explained by a so-called ‘failure of implementation’ [33]. Nevertheless, it is noteworthy that more nurses from the team and leaders-directed group completed the knowledge quiz compared to nurses from the state-of-the-art group (37% and 13%, respectively; p = 0.001). A possible explanation is that the team and leaders-directed strategy positively influenced the adherence to specific components of the state-of-the-art strategy.

### Effects of contextual factors on nurses’ HH compliance

#### Hospital culture

The as-received analysis showed a hospital effect that was mainly due to one hospital. Especially in the long run, HH compliance started to decrease in this particular hospital, while HH compliance in the other two hospitals remained stable or increased further. Little is known about how hospital cultural factors are associated with the implementation of HH improvement strategies. The WHO [3], Larson, et al. [34] and Pittet [23] emphasize the commitment of high-level administrators to create and support a culture of safety and accountability. Culture manifests itself through the values, beliefs and assumptions embedded in organizations and is reflected in ‘the way things are done around here’ [35]. The two hospitals that showed sustainability in HH compliance designated HH as a hospital-wide priority. The third hospital was less explicit and distinct in addressing the goal of HH as an organizational priority. This raises the question of whether the observed changes in HH compliance were affected by hospital culture.

### Standard care activities

Although the average HH baseline scores of the wards were comparable between wards from both groups, our analysis showed that a high baseline HH compliance was associated with a smaller effect of both HH improvement strategies. High HH compliance at baseline was particularly seen in the paediatric wards. Wagner and Kanouse [36] have pointed out that standard care activities may affect adherence behaviours and thus intervention outcomes. It is possible that certain components of our improvement strategies are already part of daily practice in some wards and therefore leave less room for improvement. Despite the influence of baseline scores and hospital effect, the team and leaders-directed strategy significantly contributed to an additional increase in nurses’ HH compliance, both short and long term.

### Effects of experiences with the improvement strategies on nurses’ HH compliance

The exploration of the relation between determinants of success and HH compliance provided empirical evidence for performance feedback, social influence and leadership as important vehicles for changing HH behaviour. It seems likely that the mixture of these strategy components affect the teams’ abilities to focus on achieving their HH improvement goals. Our results have strengthened the theoretical underpinning of the composition of our team and leaders-directed strategy by using a team approach for changing individual behaviour. By setting clear norms and targets within the team, individual team members are invited to support each other in achieving this goal.

### Speak up

The findings of our study also show that it is important to promote a team culture that empowers team members to speak up when non-adherence is observed. In this finding, we recognize key elements from the social influence theory [27] (e.g., team members address each other in case of undesirable behaviour), and the theory on team effectiveness [28,29] (e.g., participation safety and task orientation) (Table 2). This is of particular interest because ‘speak up’ is positively correlated with improved HH behaviour. During the team sessions, we taught the nurses to provide feedback on the HH behaviour of their colleagues in a correct way. At the same time, we guided the nurses to receive this feedback positively.

### Active commitment and initiative from ward management

The results of our study show that specific components of leadership are positively correlated with an improvement in nurses’ HH compliance. Thus, ward managers should address barriers to enable HH as recommended, designate HH as a ward priority, motivate and encourage team members to perform HH, and hold team members accountable for their HH behaviour. This finding corresponds with the key elements from theory of leadership [30] as displayed in Table 2.

Credits of our findings are not entirely due to the delivery of the team and leaders-directed strategy. Nurses from the state-of-the-art group were not exposed to social influence and leadership as a result of improvement activities from our study. A possible explanation is that these wards, independent of our study activities, have given priority to HH and were motivated and encouraged by their managers. This explanation is supported by the results of a further analysis within the group of the state-of-the-art strategy. We found a significant relation between changes in HH compliance and differences in nurses’ experiences with social influence and leadership. Compared to the state-of-the-art group, the analysis within the group of the team and leaders-directed strategy showed less variation in changes of nurses’ HH compliance. Therefore, an association between changes in HH compliance and differences in nurses’ perceptions of strategy components within the team and leaders-directed group could not be demonstrated. We hypothesize that the lack of variation in this group is due to the consistent implementation of the team and leaders-directed strategy. As already shown by our evaluation of strategy adherence, all nurses within the group of the team and leaders-directed strategy were equally exposed to the main components of this strategy.

### Strengths and limitations

The principal strength of our study was the comprehensive process evaluation within the context of a pragmatic randomised controlled trial. Questions about variations in the adherence to both HH strategies, and about factors contributing to the relationship between the HH improvement strategies and nurses’ HH outcomes, would not have been apparent as a result of only analysing the HH outcome data. Process evaluations are, in this sense, part of a more theory-based approach to evaluation, responding to the need to understand which theoretical constructs of an improvement strategy make a difference [37]. By linking data of effectiveness to process data, a theoretical explanatory model can be derived from the process evaluation itself [36].

Some researchers encourage the simultaneous application of a process evaluation in control groups [5,38]. By doing so, we discovered the impact of specific aspects of social influence and leadership in the state-of-the-art group that served as a control group. This finding has strengthened the theoretical underpinning of the composition of our team and leaders-directed strategy.

In combining process with outcome evaluations, we collected data using a wide range of methods as recommended by several authors [5,15]. We developed a questionnaire, derived from the components of the improvement strategies. We undertook extensive pilot work to ensure that all important components of the strategies were adequately captured in questionnaire measures. We then pre-tested the questionnaire among 90 nursing students.

An important issue concerns the use of ‘as-received’ analysis as distinct from the conventional ‘intention-to-treat’ analysis used in the analysis of RCTs. These analyses differ not only in terms of the estimation procedure, but also in terms of the underlying research goal for a specific study. This study is an example of explanatory research, and the as-received analysis was therefore appropriate. Our as-received analysis was illuminating but also lost the benefits of the original random assignment, and therefore the potential for bias exists. This should be considered when interpreting our results [39].

A limitation of our study concerns the low questionnaire response rate of 48%. This may be a potential source of bias. We didn’t test the psychometric properties of the questionnaire. For these reasons, our findings from the nurses’ experiences analysis need to be interpreted with caution.

### Implications

This is the first prospective study that has assessed the working mechanisms of two HH improvement strategies, demonstrating the added value of specific aspects of social influence and leadership. This is an important finding for hospital administrators and ward managers who want to improve nurses’ HH behaviour. Currently, most strategies focus on the individual and the organization. Including activities aimed at social influence and leadership could be a promising development. Our results point to: addressing each other in case of undesirable behaviour, support from colleagues, accountability, goal setting, and active commitment of the ward manager. The methodology of our team and leaders-directed strategy can probably be used to improve team performance on other patient safety issues as well.

Our study points to ways in which the design of process evaluations within randomised controlled trials may be conducted. Our initial results require affirmation by further process evaluations of HH improvement strategies. Further research is also needed to examine the different aspects and impact of social influence and leadership. Finally, future research should explore the influence of hospital culture.

## Conclusion

In summary, with this study we were able to look inside the ‘black box’ of two HH improvement strategies, to generate insights into which strategy components are effective. Our results support the added value of social influence and enhanced leadership in HH improvement strategies, thus offering an interpretation of the trial effects. Our findings point to: addressing each other in case of undesirable HH behaviour, support from colleagues, accountability, goal setting, and active commitment of the ward manager. These results have strengthened the theoretical underpinning of the composition of our team and leaders-directed strategy. Our study also points to ways in which the design of process evaluations within randomised controlled trials may be conducted.

### Ethical and legal aspects

The Medical Ethics Committee of district Arnhem – Nijmegen assessed the study and concluded that our study was deemed exempt from their approval, as it did not include collection of data at the level of patients.

## Competing interests

All authors have completed the Unified Competing Interest form at http://www.icmje.org/coi_disclosure.pdf (available on request from the corresponding author) and declare: no support from any organization for the submitted work; no financial relationships with any organizations that might have an interest in the submitted work in the previous three years; no other relationships or activities that could appear to have influenced the submitted work.

## Authors’ contributions

T van A, LS, MH and RG were responsible for the research question and the design of the study. AH and GH designed and operationalized the team and leaders-directed strategy. AH conducted the study, prepared and coordinated the field observations, and did the data analysis. AH wrote the first draft of this manuscript and was responsible for the revisions. T van A, LS, RG, GH, and MH contributed to the drafting of the manuscript. T van A is the general supervisor of the study. All authors read and approved the final version of the manuscript.

## Source of funding

This study is funded by a research grant from ZonMw, dossier number: 94517101.

## Supplementary Material

Additional file 1 Questionnaire on nurses’ experiences with strategy components. Click here for file

Additional file 2 Adherence of nursing wards to strategy components. Click here for file
