# Supplementary material for: Explaining the effects of two different strategies for promoting hand hygiene in hospital nurses: a process evaluation alongside a cluster randomised controlled trial
Source: Implement Sci. 2013 Apr 8;8:41. doi: 10.1186/1748-5908-8-41 (PMC3646709; doi:10.1186/1748-5908-8-41)
Supplement: Additional file 1 — Questionnaire on nurses’ experiences with strategy components. [file 1748-5908-8-41-S1.pdf]

Additional file 1: questionnaire on nurses experiences with strategy components

	Agree			Disagree
When I am wearing gloves, I don’t have to perform hand hygiene	☐	☐	☐	☐
Sinks are awkwardly placed at my ward	☐	☐	☐	☐
My colleagues think that the hand hygiene prescriptions do not always need to be followed	☐	☐	☐	☐
At my ward alcohol-based hand rub is in the immediate vicinity (<1 meter) at the point of care	☐	☐	☐	☐
Hand hygiene during procedures with low risk of contamination is of less importance	☐	☐	☐	☐
Other patient safety issues are more important than hand hygiene	☐	☐	☐	☐
It often happens that soap / hand alcohol / towels or disposable gloves are not available	☐	☐	☐	☐
My colleagues support each other in performing hand hygiene	☐	☐	☐	☐
Not performing hand hygiene could have (severe) implications for the patient	☐	☐	☐	☐
My manager pays regular attention to the adherence of hand hygiene guidelines	☐	☐	☐	☐
I regularly forget to perform hand hygiene	☐	☐	☐	☐
Hand hygiene is not a priority at our ward	☐	☐	☐	☐
My ward manager sets norms and targets for HH adherence	☐	☐	☐	☐
My ward manager encourages and motivates our team members to perform hand hygiene	☐	☐	☐	☐
Our team members address each other in case of undesirable hand hygiene behaviour	☐	☐	☐	☐
I know the content of the hand hygiene guidelines	☐	☐	☐	☐
I know exactly when to perform hand hygiene	☐	☐	☐	☐
It’s important to perform hand hygiene during procedures with high risk of contamination	☐	☐	☐	☐

I know exactly how to perform hand hygiene	☐	☐	☐	☐
Infection prevention is an important topic on my ward	☐	☐	☐	☐
I do know my wards hand hygiene performance	☐	☐	☐	☐
My ward manager provides resources to enable hand hygiene as recommended	☐	☐	☐	☐
My ward manager addresses barriers to enable hand hygiene as recommended	☐	☐	☐	☐
My ward manager holds team members accountable for hand hygiene performance	☐	☐	☐	☐
