# Supplementary material for: Explaining the effects of two different strategies for promoting hand hygiene in hospital nurses: a process evaluation alongside a cluster randomised controlled trial
Source: Implement Sci. 2013 Apr 8;8:41. doi: 10.1186/1748-5908-8-41 (PMC3646709; doi:10.1186/1748-5908-8-41)
Supplement: Additional file 2 — Adherence of nursing wards to strategy components. [file 1748-5908-8-41-S2.pdf]

Additional file 2. Adherence of nursing wards to strategy components

**State-of-the-art group *n*=47**		
Component	***Improvement activities***	***Adherence***
Education	Presence educational website and knowledge quiz (*content)*	100%
	Participation in knowledge quiz (*coverage)*	11%
	Presence of leaflets (*content)*	100%
	HH promotion event (*content)*	68%
Reminders	Three newsletters to ward manager (*content)*	100%
	Publication in hospital magazine (*content)*	100%
	Distribution of hand hygiene posters twice (*content)*	100%
	Presence of hand hygiene posters on the wards *(coverage)*	100%
Performance feedback	Distribution performance feedback reports to ward manager twice (*content)*	100%
Facilities and products	Presence of hand hygiene products (*content)*	100%
	Acceptable access to washstands / hand rub *(coverage)*	45%
**Team and leaders-directed group *n*=20**		
Component	***Improvement activities***	***Adherence***
Education	Presence educational website and knowledge quiz (*content)*	100%
	Participation in knowledge quiz (*coverage)*	37%
	Presence of leaflets (*content)*	100%
	HH promotion event (*content)*	75%
Reminders	Three newsletters to ward manager (*content)*	100%
	Publication in hospital magazine (*content)*	100%
	Distribution of hand hygiene posters twice (*content)*	100%
	Presence of hand hygiene posters on the wards *(coverage)*	100%
Performance feedback	Distribution performance feedback reports to ward manager twice (*content)*	100%
Facilities and products	Presence of hand hygiene products (*content)*	100%
	Acceptable access to washstands / hand rub *(coverage)*	40%

Setting norms and targets	Team discussion organised (*content)*	100%
	Number of team discussions *(dosage)*	92%
	Nurses' participation in team discussions *(coverage)*	50%
	Time spent on team discussions (*dosage)*	90%
	**Topics**<br>Goal setting in hand hygiene performance *(content)*	100%
	Analysis of barriers and formulating improvement activities *(content)*	100%
	Norms and targets established *(coverage)*	100%
Social influence	Nurses address each other in case of undesirable hand hygiene behaviour *(content)*	100%
Leadership	Ward manager discusses hand hygiene compliance rates with team members *(content)*	95%
	Ward manager designates hand hygiene as a priority *(content)*	95%
	Ward manager actively supports team members and informal leaders *(content)*	95%
Modelling	Informal leaders model social skills of team members in addressing hand hygiene behaviour of colleagues *(content)*	90%
	Informal leaders demonstrate good hand hygiene behaviour *(content)*	90%
	Informal leaders instructs and stimulates colleagues in providing good hand hygiene behaviour *(content)*	90%
